# Application of Positron Annihilation Spectroscopy in Accelerator-Based Irradiation Experiments

**DOI:** 10.3390/ma14216238

**Published:** 2021-10-20

**Authors:** Vladimir Krsjak, Jarmila Degmova, Pavol Noga, Martin Petriska, Stanislav Sojak, Matus Saro, Igor Neuhold, Vladimir Slugen

**Affiliations:** 1 Slovak University of Technology, Faculty of Electrical Engineering and Information Technology, Institute of Nuclear and Physical Engineering, Ilkovicova 3, 81219 Bratislava, Slovakia; jarmila.degmova@stuba.sk (J.D.); martin.petriska@stuba.sk (M.P.); stanislav.sojak@stuba.sk (S.S.); matus.saro@stuba.sk (M.S.); igor.neuhold@cern.ch (I.N.); vladimir.slugen@stuba.sk (V.S.); 2 Slovak University of Technology, Faculty of Materials Science and Technology, Advanced Technologies Research Institute, Jana Bottu 2781/25, 91724 Trnava, Slovakia; pavol.noga@stuba.sk

**Keywords:** ion irradiation, positron annihilation spectroscopy, positron lifetime, Doppler broadening spectroscopy, f/m steels, RPV steels, oxide dispersion strengthened steels

## Abstract

Positron annihilation spectroscopy (PAS) is widely recognized as a powerful characterization technique in all types of radiation damage studies in nuclear materials. In the past, fission reactor irradiation of reactor pressure vessel (RPV) steels was a primary aim in most studies, while today’s applications of PAS in this field are centered around ion implantation experiments in advanced structural materials. These experiments use hydrogen, helium, heavy ions, and their combination to simulate various radiation environments of future nuclear reactors or nuclear research facilities. The spectrum of ion energies used ranges from a few tens of keV to tens or even hundreds of MeV in proton irradiation or spallation neutron source irradiation experiments. The variety of ion energies, irradiation temperatures, and other experimental conditions poses a major challenge to researchers, who often fail to successfully incorporate the lessons learned from their research. In this paper, we review and supplement recent PAS studies in which structural materials irradiated under a variety of irradiation conditions were investigated using positron annihilation spectroscopy. It summarizes the most important conclusions and lessons learned from the application of PAS in accelerator-based irradiation experiments.

## 1. Introduction

The experimental simulation of the harsh radiation environments of future nuclear fission and fusion reactors requires compromises to be made on the selection of bombarding particles and parameters such as flux, energy spectrum, or the production of transmutation elements and the change in the chemical composition of the target material. Additionally, for the characterization techniques employed, it is inevitable to consider the shape of the damage profile, particularly when using low to intermediate energy (tens of keV to hundreds of keV) of charged particles.

Among the numerous analytical techniques used in material irradiation studies [1,2,3], positron annihilation spectroscopy (PAS) is well known for its spectacular sensitivity to atomic-scale vacancy-type defects, and it has been widely used in the past [4]. This unique sensitivity originates from the fact that positrons are attracted to regions of the lattice with an open volume. The positron, unlike any other particle, acts as a self-seeking probe for vacancy-type defects in condensed matter. Due to its unique features and non-destructive nature, PAS has been recognized as a convenient complementary tool for the microstructural characterization of lattice defects.

The published data refer primarily to irradiation studies of reactor pressure vessel (RPV) steels [5,6]. Despite being a valuable contribution to understanding radiation-induced degradation processes in these materials, the lessons learned cannot be simply extrapolated to the new challenging radiation conditions of future nuclear fission and fusion reactors [7]. The use of fast neutron irradiation experiments to simulate the harsh radiation environments of future reactor systems faces two significant hurdles. While it provides reasonable displacement damage rates in producing collision cascades, it does not provide an adequate simulation of gaseous transmutation products, such as hydrogen or helium, severely impairing the defect recombination processes in the displaced matrix. The second, obvious complication arises from the induced activity of the neutron-irradiated samples; the handling of which requires dedicated nuclear facilities and radioisotope laboratories. Both issues can be conveniently solved by using ion implanters in either single-beam or dual-/triple-beam configurations. Various PAS studies of materials modified by ion bombardment, in a wide range of ion energies and fluences, have been published in the last decade. In addition to the structural materials addressed in this paper, plasma-facing materials [8,9,10,11] and nuclear fuel [12,13,14] have been investigated using this approach.

Comparably to ion irradiation experiments, slow positron beam experiments enable the region of interest within the studied sample to be precisely selected. This makes positron beam techniques natural approaches to studying accelerated radiation damage in materials. Most of the studies use slow positron beams with positron energy of up to a few tens of keV, corresponding to a mean implantation depth in the micrometer range in most nuclear-relevant materials. These studies have generally been aimed at ion implantation experiments using accelerating energies of up to a few MeV. There have been very few irradiation studies published on nuclear structural materials utilizing charged particle irradiation of the target at energies of 10–100 MeV [15,16,17]. An example of such an implantation (multi-energy He^+^ implantation of structural steels) is discussed in the paper by Noga et al. in this Special Issue. Moving up with the particle accelerator energy, the irradiation experiments necessarily cause activation of the material; for instance, by spallation reactions [18]. This radiological complication is, however, outweighed by extremely interesting irradiation data, involving realistic and fusion-relevant displacement damage rates, helium and hydrogen production rates, as well as quasi-homogeneous damage distribution over a bulk sample [19]. Concerning PAS, the last feature mentioned enables the convenient use of radioisotope positron sources with a continuous energy spectrum to probe the irradiated material. The comparison between conventional (radioisotope-based) PAS techniques and slow positron beam techniques is discussed in the next chapter.

While the individual PAS techniques provide excellent reproducibility of the results in various studies of semiconductors and non-metallic solids, the results obtained from the investigations of realistic alloys exposed to dissimilar irradiation conditions can rarely be found to correlate between different research studies. Here, let us omit the fundamental distinction between the near-surface studies, utilizing slow positron beams, and the “bulk” studies, based on unmoderated radioisotope positron sources. Quite substantial discrepancies between the acquired results can arise from the same kind of post-irradiation PAS examination. There are several potential reasons for this, which can be divided into two categories. The first group of issues relates to the apparatus, signal processing, and data processing. In this regard, particular attention must be paid to suppressing the noise signal originating either from transmutation elements or Compton-scattered positron annihilation gamma. While three-detector positron lifetime spectrometers [20] and a coincidence setup of Doppler broadening spectrometers [21] can potentially solve this problem, the deviations in the geometry and activity of the used positron sources, as well as in the isotopic composition of the measured samples, affect the effectivity of this solution. Another source of discrepancy between different PAS characterizations, for instance, ion-implanted samples, comes from the lack of consensus in the data evaluation. While some studies report the peak values of displacement damage and ion concentration data, others report an integral value over the whole implantation profile, or an integral value over the probing particle stopping profile. It is important to note that the stopping profile of a monoenergetic positron is very broad at high-incident energies (tens of keV), and while the mean stopping depth of the positron can be accounted for the peak region of the ion-modified layer, a substantial amount of signal can come from either the substrate or the thermal spike region.

The first and foremost benefit of using PAS techniques in nuclear materials irradiation experiments is that it allows the detection of the slightest changes in the microstructure, associated either to defect production or defect recombination processes. Positron annihilation characteristics enable a more comprehensive interpretation of conventional TEM analyses and the results obtained from mechanical testing. To produce relevant and reliable complementary information to conventional imaging and destructive methods, numerous aspects need to be considered. This paper addresses the application of PAS techniques in the characterization of materials exposed to different types of irradiation experiments. It reviews recent studies in the field, and provides some empirical support for exploiting the full potential of positron annihilation spectroscopy in nuclear material research.

## 2. Methodology

Positron annihilation spectroscopy (PAS) refers to a qualitative and quantitative analysis of spectra acquired from different spectrometry techniques. The two most common techniques based on electron-positron annihilation are positron annihilation lifetime spectrometry (PALS) and Doppler broadening spectrometry of annihilation radiation (DBS). It is important to note that an overwhelming number of published studies, including the present study, refer to spectroscopy rather than spectrometry. The likely reason for this is that the aim of these studies is usually focused on the origin and properties of the acquired spectra rather than on the actual measurement. The unique feature of the positron as a probe for microstructural characterization is its remarkable sensitivity to crystal lattice defects, ranging in size from monovacancy to open-volume defects or precipitates of few nanometers in diameter. How efficiently the defect acts as a potential well for the positron depends mostly on the size of the defect (open-volume defects) and the positron affinity (precipitates—how different the positron affinity is to that of the matrix). The accuracy of determining the interaction volume of the positron depends on its energy. The selection of a suitable PAS technique depends on the actual phenomenon studied.

### 2.1. Positron Stopping in Solids—Interaction Volume

The availability of different radioisotope sources and variable-energy slow positron beams enables the positron annihilation-based experiments to be optimized to suit the material damage profile to be investigated. Although studies focusing on a particular type of positrons source and corresponding stopping profile have been published in the past [22,23,24,25], empirical studies employing both radioisotope positrons and slow positron beams in the same field of material research are rather scarce. In the material research studying radiation effects, the radioisotope source has been used typically in the context of neutron irradiation and gamma irradiation, i.e., deeply penetrating ionizing radiation. On the contrary, slow positron beams have been predominantly used in the studies employing charged particle accelerators, producing a typical narrow-peak damage profile in the irradiated samples. With the increasing energy of particle accelerators and availability of high-intensity ion beams of tens of MeV, the classical dividing between “near-surface” and “bulk” radiation damage studies loses its justification. In the following section, we summarize some fundamental knowledge on the interaction volume associated with positron stopping in solids.

#### 2.1.1. Radioisotope Positron Sources

Beta-plus decay radioisotopes are the most common types of positron sources used in material research. In addition to the requirement of a reasonably long half-life (at least a few years), the emission of the positron must be accompanied by the emission of characteristic gamma to enable the application of techniques based on positron lifetime measurements. It is important to note that regardless of the source design, a fraction of positrons will not escape from the source, and electron-positron annihilation signals will always contain a source contribution. The most common procedure for positron source preparation is based on the use of ^22^NaCl solution evaporated and sealed in a Kapton encapsulation. Depending on the thickness of the Kapton foil and the effective diameter, the source contribution of such sources can vary from ~7 to ~20% [26,27].

**Figure 1:**
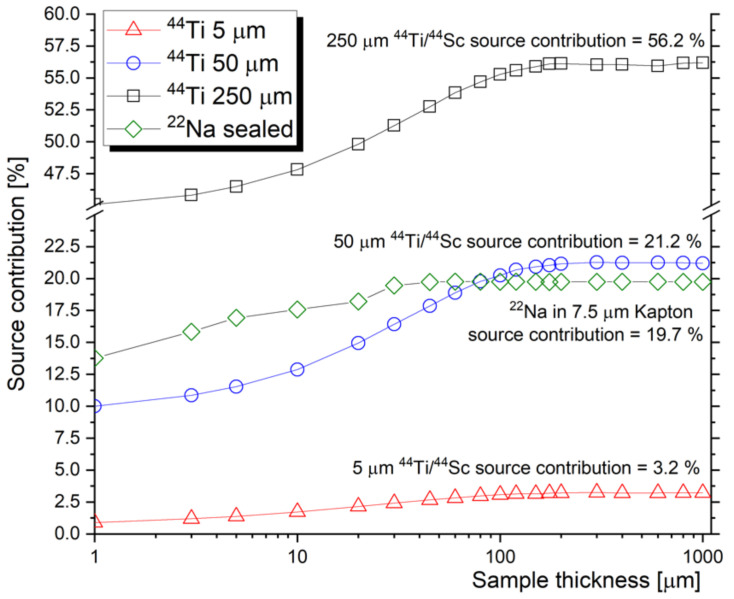
GEANT4 simulation of the source contribution for ^44^Ti/^44^Sc (different thickness of the source foil) and ^22^Na (encapsulated in 7.5 μm Kapton foil).

Another type of positron source with various prospective applications is based on a ^44^Ti/^44^Sc radionuclide generator. Due to the beneficial mechanical properties of metallic foils, compared to brittle ionic bonds in ^22^Na sources, no encapsulation is required to prevent sample contamination from the positron source. Since the production of ^44^Ti requires a high-energy proton implantation [28] with limited availability of the information about actual reactions and reactions cross-sections, it is necessary to consider the contribution of positron annihilation inside the source as a function of its thickness. The actual source contribution is, however, also a function of the fraction of backscattered positrons. In the field of nuclear materials and testing of miniaturized samples, it is reasonable to outline the fraction of backscattered positrons as a function of the tested sample’s thickness. Figure 1 shows the ^44^Ti/^44^Sc source contribution calculated using GEANT4 code [29] for three different source thicknesses and various thicknesses of the used sample (Fe). With a sufficiently thick sample, the (saturated) source contribution was estimated to be 3.2%, 21.2% and 56.2% for the titanium/scandium source in the form of a 5 μm, 25 μm and 250 μm thick foil, respectively. In the case of thinner samples, the contribution of the source is reduced by the fraction of positrons escaping the source-sample system (no backing materials considered in the simulations). Figure 1 also includes a commonly used positron source based on ^22^Na encapsulated in 7.5 μm Kapton foil.

**Figure 2:**
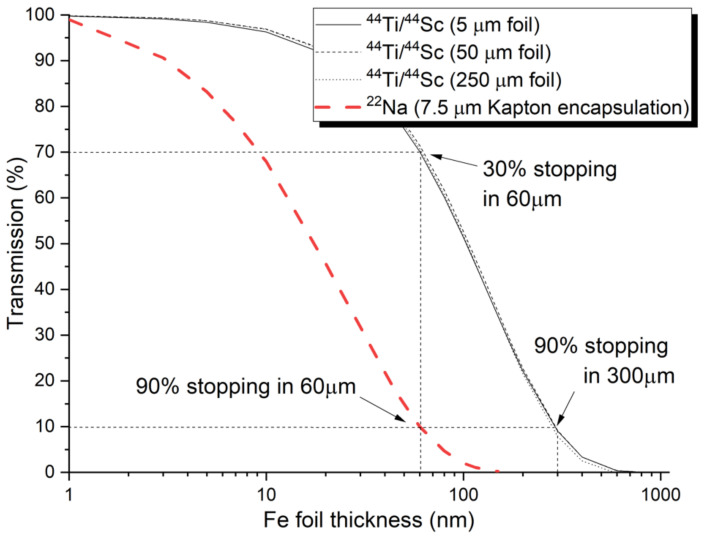
Stopping profile of positrons emitted from a ^22^Na Kapton-encapsulated source (thick dashed line) and from ^44^Ti/^44^Sc foil sources of different thicknesses (thin lines).

Although the energy spectrum of positrons (i.e., the positron stopping profile) from any source depends on the encapsulation and the actual thickness of the source, positron mean stopping depth from the ^44^Ti/^44^Sc source is roughly 5× deeper than the stopping depth of ^22^Na positrons. Figure 2 shows the estimated stopping profile of positrons from the two radioisotope sources in Fe. While the ^22^Na source that is shown is encapsulated in Kapton, the ^44^Ti/^44^Sc is considered in the form of metallic foil with different thicknesses. The results are obtained from GEANT4 simulation for 7.5 μm Kapton-encapsulated ^22^Na source and ^44^Ti/^44^Sc source. Note that the thickness of the source does not significantly moderate the energy spectrum, and the stopping profile of the positrons from different thicknesses of titanium foil is practically identical.

The choice of optimal source depends on the nature of the positron characterization experiments as well as on the investigated material. While metallic sources are usually preferred for semiconductors studies, polymer-encapsulated sources provide a good choice for studies on metallic samples. The study by Saro et al. [30] concludes that practically measurable signal (20% positrons stopped in the modified region) from ion-implanted region can be obtained for ion beam modified layer of at least 3 μm thickness. This is a practical reference for mechanical surface treatment where the layer of plastically deformed material can reach up to 1 micrometer even when sub-micrometer diamond polishing slurry is used [31]. Therefore, electropolishing following careful mechanical polishing is strongly suggested as a surface preparation procedure for any experiments involving moderated or slow positron.

Several papers have been published on analytical characterization of positron stopping profile in different metals with a good correlation in the bulk region and certain discrepancies in the near-surface region [27,32]. These discrepancies may originate from low-energy positron backscattering (function of Z) and the actual design of the considered positron source.

#### 2.1.2. Slow Positron Beam Experiments

A relatively wide interaction range of electron-positron annihilation in experiments utilizing radioisotope positron sources is not practically suitable for narrow ion beam modified layers. Various techniques based on slow positron beams have been developed to study thin multilayer systems or near-surface areas of ion-bombarded samples. While the positron yield of the sources in such facilities is below the yield of conventional radioisotope positron sources, there have been some high-intensity positron sources developed, such as the NEPOMUC source [33] at the FRMII reactor in Garching, Germany. A unique feature of such facilities is an absence of the “source component”, providing an undisturbed spectrum characterizing the studied sample exclusively.

The techniques based on slow positron beams utilize the relationship between the energy of the moderated positron beam and the depth of the positron stopping. The mean value of the depth of the positron stopping profile, $\bar{z}$, is a function of the positron energy and target material density ρ (Equation (1)).

(1) $$\bar{z}\left[\mathrm{cm}\right]=\frac{AE{\mathrm{keV}}^{n}}{\rho \left[g\;{\mathrm{cm}}^{-3}\right]}$$

The constants A = 40 ${g\;\mathrm{cm}}^{-2}{\;\mathrm{keV}}^{-n}$ and *n* = 1.6 are empirically determined and applicable for a wide range of structural materials. The shape and width of the monoenergetic stopping profile can be described by the Makhovian formula (probability density function (2)), where $z_{0}$ is related to the mean implantation depth by $z_{0}=\frac{2\bar{z}}{\sqrt{\pi }}$ and the shape parameter m = 2 [34].

(2) $$P\left(z,\;E\right)=\frac{mz^{m-1}}{z_{0}^{m}}e^{-{\left(\frac{z}{z_{0}}\right)}^{m}}$$

**Figure 3:**
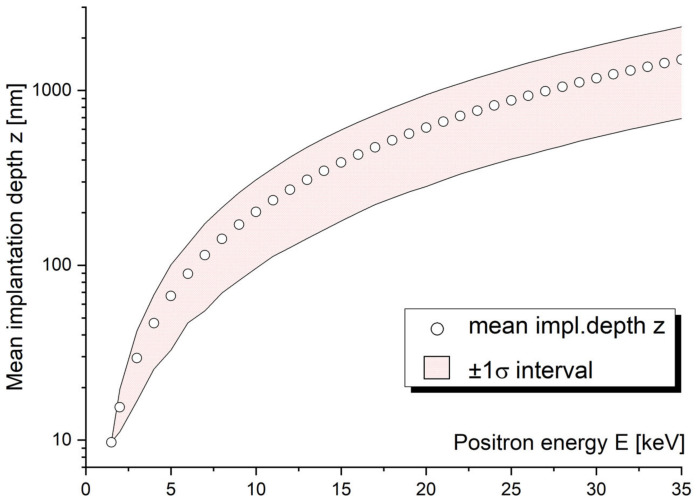
Mean implantation depth of positrons with variable energy (E). The standard deviation was calculated from the Gaussian-like Makhovian function as $\frac{FWHM}{2.3548}$, where FWHM is full width at half of the maximum [35].

While the Makhovian profile is relatively narrow for low positron energies (<10 keV), it broadens significantly for energies above ~15 keV, as can be observed in Figure 3 (note: log scale on y-axis). This a very important feature to be considered in the interpretation of slow positron beam data obtained on multilayer samples or ion-implanted samples; for instance, one sigma interval corresponding to Makhovian profile of 15 keV positron is 200 nm, with a mean implantation depth of 440 nm.

### 2.2. Techniques of Positron Annihilation Spectroscopy

Various techniques of positron annihilation spectroscopy have been established in the past to provide unique information at the atomic scale on characterizing defects in crystalline materials both in qualitative and quantitative terms. The two most common techniques are positron annihilation lifetime spectroscopy (PALS) and Doppler broadening spectroscopy of the annihilation gamma peak (DBS) [36]. While the fundamental principles of the two techniques (described in detail below) are different, a good correlation can often be obtained by their complementary application [37]. Together with transmission electron microscopy (TEM) and some other microstructural characterization methods, such as SANS and Raman spectroscopy, PAS provides solid support for interpreting the results obtained from micromechanical testing methods.

#### 2.2.1. Positron Annihilation Lifetime Spectroscopy (PALS)

Positron annihilation lifetime spectroscopy is a widely used analytical technique utilizing the phenomenon of positron trapping by defects and the lifetime of the positron being dependent on the nature and size of this defect. The values of bulk lifetimes and elementary defects are well known virtually for all basic metals and simple alloys, enabling a qualitative characterization of various defects introduced in irradiation, thermal or mechanical treatment. There are a few practical limitations of qualitative analyses by positron lifetime spectroscopy. First and foremost, the limited number of components into which the lifetime spectrum can be decomposed. There are rarely more than 2–3 discrete components determined, additional to the ones assigned to the source contribution. This problem is even more pronounced in experiments leading to combined defects (e.g., jogs or vacancies on dislocation lines) or a broad continuous spectrum of open-volume defect size (instead of a sharp unimodal or bimodal distribution) complicating the spectra decomposition. Although modern digital lifetime spectrometers [38] have excellent time resolution (FWHM < 150 ps), reliable decomposition requires a difference of at least several tens of ps in lifetimes of the identified components. Finally, it is essential to consider all contributions to the lifetime spectra outside the source sample sandwich, such as sample holder or positron backscattering to sample surface (or any internal surface), which act as a strong trapping site. The experiment geometry and good calibration samples are essential in this regard.

**Figure 4:**
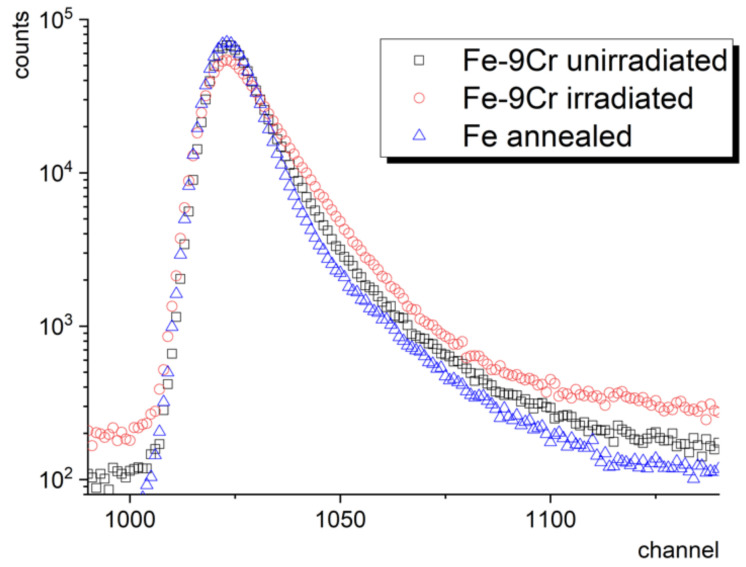
Lifetime spectrum of defect-free annealed Fe, together with the spectrum of Fe–9Cr steel before and after exposure to radiation environment of spallation neutron source [37].

Figure 4 shows a typical 1M count lifetime spectrum of a ferritic/martensitic (f/m) steel sample in as-received condition and after irradiation in spallation neutron source to 20 dpa (2000 appm He) [37]. Besides, the figure shows the lifetime spectrum of pure defect-free well-annealed Fe, i.e., a two-component (Fe sample + source encapsulation) lifetime spectrum. One can observe a distinct increase in background as well as an increase in the contribution of longer lifetime components in the irradiated Fe–9Cr sample. This example shows a particular case when the trapping of radiation-induced defects leads to near-saturated positron trapping when the whole lifetime spectrum can be described by a single component (fitted by a single exponential curve) and the short lifetime component cannot be identified anymore. For more details on this, view the positron trapping model in Section 2.4.

#### 2.2.2. Doppler Broadening Spectroscopy

Spectroscopy based on the measurement of the Doppler broadening of the annihilation peak is based on the momentum conservation law of the electron–positron pair and the fact that the contribution of the positron to this momentum is practically negligible. This enables us to observe the changes in the shape of the annihilation peak induced by changing ratio between low-momentum valence/conduction electrons and high-momentum core electrons. It also enables changes in the atom-specific core electron distribution to be detected and thus provides chemical information from the vicinity of the annihilation site.

**Figure 5:**
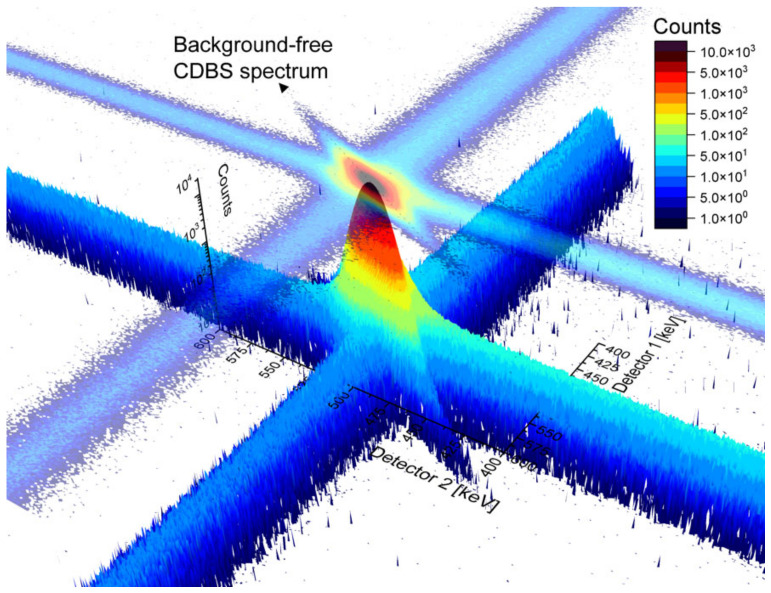
CDBS spectra obtained on defect-free Fe in a three-dimensional plot and two-dimensional projection. The elliptical region extending diagonally with E_D1_ + E_D2_ = 1022 keV is virtually background free.

In PALS spectra, the lower count region corresponds to a long positron lifetime, often not very meaningful in the research of metals and alloys, while the low count region of the Doppler-broadened annihilation line is very important for the investigation of the chemical fingerprint of the annihilation site. The wing parts of the spectra describe the contribution of core electron annihilations and, in a simple one-detector DBS spectrometer (gamma spectrometer), remain almost entirely masked by the background. The reduction in the background and increase in the peak-to-background ratio is therefore very important. A convenient, well-established way to obtain virtually no background DBS profile is to use two HPGe detectors facing opposite sides in a coincidence setup. This only enables the recording of events when two (annihilation) gammas are detected in a certain time window, i.e., time discrimination. The spectra of such a coincidence setup of DBS, referred to as CDBS, are recorded in the form of a two-dimensional matrix, which can be plotted as a three-dimensional image, as shown in Figure 5. The analysis of the spectra is performed using a diagonal indicated in the two-dimensional projection of the spectra.

**Figure 6:**
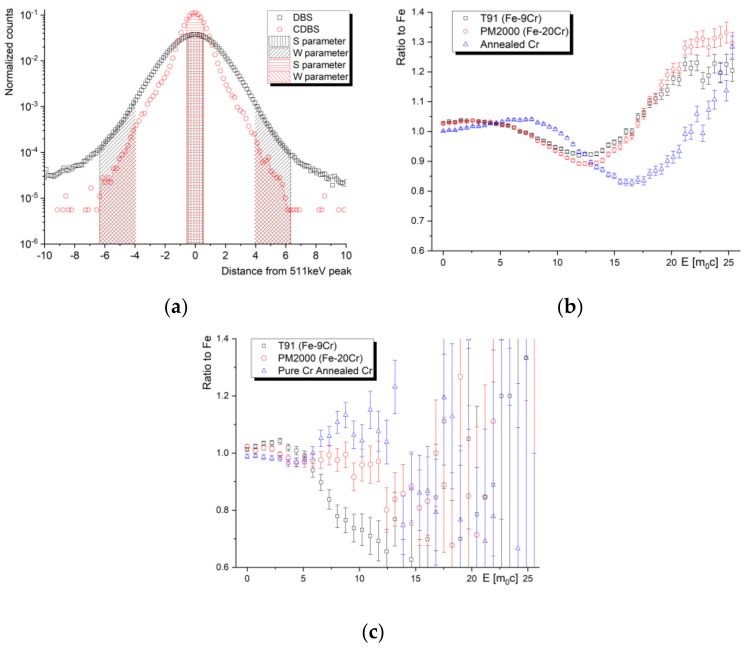
Normalized DBS spectra (**a**) of pure Fe obtained from CDBS matrix as the main diagonal (referred to as DBS) and as the sum of the lines (columns), respectively (referred to as CDBS). DBS ratio curves, relative to Fe, of two steels with different Cr content and pure Cr sample obtained for the first case (**b**) and for the second case (**c**).

Naturally, the count rate in 2D CDBS spectra is significantly (roughly an order of magnitude) lower compared to single-detector DBS spectra. The counts determine the actual time needed to acquire a spectrum in the high-momentum (low count) region. Our recent experiments on thermal-induced Cr precipitation in PM2000 steel, however, show that the best compromise between the sensitivity to the chemical changes in the microstructure and the count rate can be obtained from the 2D projection of the spectra to the X(Y)Z plane, i.e., when the coincidence spectrum is discriminated with respect to the time (the event detected by first detector is considered valid only when the second detector detects a signal within the given time window). In other words, the “massive” reduction in count rate in the diagonal region of the CDBS spectra is not always outweighed by the benefits of “massive” background reduction. Figure 6a shows normalized one-dimensional Doppler broadening spectra obtained as the main diagonal of the CDBS matrix (10^7^ counts) and as the sum of the lines (columns), respectively. One can clearly observe an increased peak-to-background ratio in the main CDBS diagonal. On the other hand, the amount of counts in the summed lines (columns) is significantly reduced in the high-momentum region. This leads to a significant worsening of the uncertainty, calculated as 1/sqrt(counts), in this region. Figure 6b,c show DBS momentum curves relative to the pure Fe curve obtained for the two discussed cases. In spite of different uncertainty given by different statistics of counts, the shape of the ratio curves also varies between different data treatment procedures, as can be observed from the figures. Chemical interpretation of the CDBS data obtained on complex engineering materials, such as alloys and steels, must be performed with caution. It is important to note that more sophisticated CDBS spectra treatments are often used in similar studies, improving the uncertainty of the acquired data. It is, nevertheless, beneficial to consider the setup of the equipment, data treatment procedure and the target statistics of the spectra. Meaningful Doppler broadening data can be obtained from a coincidence setup utilizing one HPGe detector and one (much more affordable) scintillator detector. More detailed discussion on the processing of CDBS spectra, including reference to a new update of a CDBStools software developed at the Slovak University of Technology, can be found in a paper by Petriska et al. in this issue.

As illustrated in Figure 6a, quantification of the broadening of the annihilation peak can be performed using two line-shape parameters, S and W, which are defined as the area under the central part of the peak and the area in the “wing” sections in a background-subtracted normalized DBS curve. This approach is widely used in most of the studies aimed at the “quantification” of radiation damage in nuclear materials or efficiency of recovery annealing in irradiated materials. In the case of strong positron sources and a sufficient number of counts in the high-momentum (wing) parts of the peak, more precise insights into microstructural changes can be obtained by evaluation of the actual momentum curves plotted as a ratio to the reference sample, as illustrated in Figure 6b,c. In the case of nuclear structural materials, the reference sample is usually a well-annealed defect-free Fe or a sample of the given studied material in the as-received condition.

### 2.3. Positron Trapping at Defects

In a perfect lattice, all positrons annihilate in the free delocalized state. The introduction of defects into the perfect lattice leads to localization of the positrons at these defects. In positron lifetime spectroscopy, it is represented by an introduction of a new (longer) defect component ($\tau_{d}$) and a reduction in the free positron lifetime, typically represented by the shortest component of the spectra ($\tau_{1}$). In the case of one type of defect, this can be explained using a simple trapping model, as follows:

(3) $$\frac{1}{\tau_{1}}=\frac{1}{\tau_{B}}+\kappa_{d}$$

where the following applies:

(4) $$\kappa_{d}\left[s^{-1}\right]=\frac{I_{2}}{I_{1}}\left(\lambda_{b}-\lambda_{d}\right)=\frac{I_{2}}{I_{1}}\left(\frac{1}{\tau_{b}}-\frac{1}{\tau_{d}}\right)$$

is the positron trapping rate at defect d, and $\lambda_{b}=\frac{1}{\tau_{b}}$ and $\lambda_{d}=\frac{1}{\tau_{d}}$ are the annihilation rates in bulk and in defects, respectively. Intensities $I_{1}$ and $I_{2}$ represent the fraction of positrons corresponding to either group. Considering a bimodal characteristic and two types of radiation-induced defects, which is a reasonable approximation, for instance, to the critical size concept of bias-driven cavity growth [39], the lifetime spectra can be analyzed in terms of three exponential components [4], as follows:

(5) $$n\left(t\right)=n_{0}\overset{3}{\underset{i=1}{\sum }}I_{i}exp\left(-\frac{t}{\tau_{i}}\right)$$

where n is the number of positrons at time t, $n_{0}$ is the number of positrons at time t=0, and $\tau_{i}$ and $I_{i}$ are the lifetimes and intensities of individual components, respectively. While $\tau_{1}$ and $I_{1}$ describe the annihilation with delocalized (untrapped) positrons, $\tau_{2},\;I_{2},\tau_{3},\;I_{3}\;$ refer to positrons trapped at defects.

In numerous works, including many of those reviewed in this paper, the (total) positron trapping rate at defects is given as a sum of the trapping rate in the defect of type 1 ($\kappa_{1})$ and the trapping rate in the defect of type 2 $(\kappa_{2}$).

(6) $$\tau_{2}=\frac{1}{\lambda_{2}}=\frac{1}{\lambda_{1}};\;I_{2}=\frac{\kappa_{1}}{\left(\lambda_{B}-\lambda_{1}+\kappa_{1}+\kappa_{2}\right)}$$

(7) $$\tau_{3}=\frac{1}{\lambda_{3}}=\frac{1}{\lambda_{2}};\;I_{3}=\frac{\kappa_{2}}{\left(\lambda_{B}-\lambda_{2}+\kappa_{1}+\kappa_{2}\right)}$$

The positron trapping rate at defects, κ [s^−1^], is directly proportional to the density of the given type of defects via the so-called positron trapping coefficient *µ* [m^3^s^−1^]. This constant has been obtained for point defect and small defect clusters in a wide range of materials. It is important to note that the trapping rate is proportional to the density of traps only in so-called transition-limited positron trapping, which assumes a small size of positron traps with a spatial extent that is much less than the positron thermal wavelength (~few nm). This assumes homogeneous distribution of (small) defects, zero probability of detraining from defects, as well as zero probability of trapping of non-thermalized positrons. In the case of large defect agglomerations (>2–3 nm), such as bubbles or voids, positron diffusion needs to be considered, and consideration of diffusion-limited trapping [40] is advised. In constant-temperature measurements (constant thermal velocity of positrons), the transition from the transition-limited to diffusion-limited regime in the irradiated samples usually arises from the lower cavity concentration and the larger trapping coefficient due to the larger cavity sizes.

In the case of more than one type of defect present in the investigated sample, it is reasonable to consider the evaluation of the average positron lifetime ($\tau_{AVG}$), rather than the lifetimes and intensities of the individual components of the spectra. $\tau_{AVG}$ is defined as a weighted average of positron lifetime components. According to the simple trapping model, with increasing concentration of defects, the positron trapping at defects is enhanced, which results in an increase in $\tau_{AVG}$.

The introduction of defects is, in terms of DBS measurements, typically accompanied by an increase in the line-shape S parameter, which characterizes the Doppler broadening of the annihilation line due to the non-zero momentum of the annihilating electrons. Unlike the positron lifetime carrying information about the local electron density at the annihilation site, the S parameter carries information about the local electron momentum distribution. This feature is particularly valuable in the case of production of transmutation inert gasses, such as helium or argon, in harsh radiation environments or in the case of radiation-induced precipitation processes.

### 2.4. Positron Diffusion in Solids

For the efficient treatment of the data obtained from the slow positron beam measurements, not only the initial implantation profile, but also the thermalized positrons diffusion should be considered. This is considered in the positron diffusion trapping model (DTM) [23].

In the case of so-called transition-limited trapping assuming homogeneously distributed (small) defects, the positron diffusion constant $D_{+}$ is not a function of space coordinates, and the effective positron lifetime $\tau_{eff}$ is proportional to the effective positron diffusion length in the given material (Equation (8)).

(8) $$L_{+,eff}=\sqrt{D_{+}\tau_{eff}}$$

$L_{+,eff}$ can be obtained from slow positron experiments utilizing monoenergetic beams of positrons with variable energy. For this purpose, the VEPFIT software [41], fitting of the curves, is traditionally used. This program numerically solves the positron diffusion equation, and provides positron diffusion lengths and ranges of individual layers in multi-layer systems. However, our recent experiment [42] on slow positron lifetime measurements suggests that the same approach can be used for $\tau_{AVG}\left(E\right)$ curves obtained from average positron lifetime data. This study builds on numerous experiments showing an excellent correlation between the $\left(E\right)$ and $\tau_{AVG}\left(E\right)$ obtained on helium-implanted steel samples.

Using Equations (3) and (8), one can express the positron trapping rate at defects [s^−1^] as a function of positron diffusion length by the following formula:

(9) $$\kappa =\frac{1}{\tau_{B}}\left(\frac{L_{+,B}{}^{2}}{L_{+,\;eff}{}^{2}}-1\right)$$

Here, $L_{+,B}$ refers to the positron diffusion length in defect-free bulk. The positron trapping rate can be obtained by Equation (9), even in the case of saturated positron trapping when the positron lifetime does not reflect the increased concentration of defects anymore. This approach is very beneficial in studies of irradiated materials with a high density of internal surfaces and other defects, such as tempered martensitic steels and nanostructured alloys.

## 3. Application of PAS Techniques in Accelerator-Based Irradiation Experiments

The following section reviews some of the recent papers published on the PAS characterization of ion-implanted structural materials used or considered in nuclear applications. More than 30 papers, selected from scientific libraries by using the keywords “positron annihilation” and “ion implantation”, have been reviewed and complemented by our studies and experiments. The presented discussion was divided into four sections, based on the type of irradiation experiment, namely, self-ion irradiation, hydrogen (proton) irradiation, helium (alpha) irradiation, and combined synergistic studies utilizing at least two of these types of ions.

### 3.1. Self-Ion Irradiation

The primary benefit of using heavy-ion or self-ion irradiation lies in the high displacement damage rate and the ability to obtain high dpa’s without a significant change in the chemical composition of the irradiated material. It is important to note that a significant fraction of the implanted damage is “lost” in the heat spikes, and the final amount of damage produced may be much less than the estimation provided by, for instance, the SRIM code [43], as this is limited to the binary collision approximation, which does not describe defect recombination. A better prediction is provided by, e.g., models based on the two-temperature model [44], with extensions focusing on the interaction with metals [45], or in combination with molecular dynamics, e.g., [46]. As in swift heavy ions, the high electronic stopping (strong inelastic electronic excitation) characterizing the initial part of the ion trajectory significantly dominates over the nuclear stopping (elastic nuclear collision). This type of irradiation experiment usually results in a relatively narrow region of ion beam modified material. From the positron annihilation perspective, the use of unmoderated radioisotope sources is practically not applicable here, and the whole damage region can be characterized by slow positron beam experiments with a positron energy of a few keV. On the one hand, the corresponding Makhovian profile corresponds to a very localized positron–sample interaction volume, while the quality of the sample surface, on the other hand, affects the obtained results significantly. Unless very careful sample preparation, finished by electrochemical polishing, is performed, the near-surface region should be described by a separate layer in the positron diffusion model. The interpretation of the positron annihilation spectroscopy data obtained on self-ion implanted materials is usually much more straightforward compared to the experiments discussed in the next chapter. Without transmutation elements, such as H or He, the positron lifetime increases monotonically with the size of the (vacancy-type) defect cluster. This enables a rather reliable qualitative characterization of the radiation-induced defects. There is also a typical linear dependency between the DBS line-shape parameters S and W, indicating one type of defect with no changes in the chemical environment at the annihilation site. The typical energy used in the self-ion irradiation experiments characterized by slow positron beam techniques, published in the literature, lies in the range of a few MeV, i.e., in the sub-μm region (Table 1).

In the work by Abhaya et al. [47], Ni^+^ ions of 1.5 MeV and fluence of 5 × 10^16^ cm^−2^ were used to produce damage of nearly 100 dpa in an FeCrCoNi high-entropy alloy at 500 °C. The irradiation led to the creation of monovacancies, which were observed to be stable up to 700 °C. The authors concluded that there was a significant effect of the grain boundaries in the polycrystalline FeCrCoNi, acting as strong sinks and preventing the formation of voids, which would be expected at the given displacement damage level and at the used irradiation temperature.

The slow positron beam experiment, utilizing both positron lifetime and Doppler broadening techniques, has been used in Agarwal et al. [48], in the characterization of a mechanism for the interaction of cascade damage with voids in Fe films. Ion irradiation of the Fe films was performed with 2 MeV Fe ions at room temperature, to a fluence of 5.65 × 10^14^ cm^−2^. The relatively low irradiation fluence (0.06 dpa in peak) resulted in the formation of a maximum of four vacancy clusters assigned to a 258 ps lifetime component. The study concludes an interesting result, where the density of small vacancy clusters increases with irradiation, whereby the size of large voids is reduced, which is an effect attributed to the high porosity of the initial microstructure. However, the authors also note the limited accuracy of defect density calculations, due to missing positron trapping coefficients for small vacancy clusters.

Another PAS study devoted to Fe, by C.W. He et al. [49], used SPB DBS to investigate the interaction between vacancies and Yttrium atoms in BCC iron irradiated with 1.2 MeV Y ions at room temperature and fluences from 1 × 10^14^ cm^−2^ to 3.0 × 10^15^ cm^−2^. The authors also observed a decrease in the size of the vacancy clusters with increasing fluence; however, in this case, the concentration of these decreased too. This effect is explained as a combination of the (i) formation of V_m_–Y_n_ complexes, as the migration temperature of the vacancies in BCC Fe is below RT and the mobility of the vacancies can be further reduced by binding with Y, which limits their agglomeration; (ii) formation of Y_m_–X_n_ precipitates (where X = Y,O, etc.), leading to a decrease in the size and concentration of vacancy clusters.

Commercial-grade 9Cr–1Mo steel (T91) was investigated by SPB DBS after implantation by 3.25 MeV Fe ions at different temperatures, by Zhu et al. [50]. The applied DBS technique revealed that the concentration of open-volume defects decreased with the implantation temperature, while the complementary nanoindentation technique showed an increase in hardness with increasing irradiation temperature. Similar irradiation conditions were used in another work of Zhu et al. [51] on the NHS, a high Si reduced activation f/m steel. In both the fluences used in this research, the S parameter was found to increase significantly after RT irradiation. On the other hand, the irradiation performed at 450 °C resulted in an S-parameter depth profile that was almost identical to the one of the unirradiated sample. This was explained by the enhanced mobility of the vacancies and interstitials at high temperatures, together with the concentration gradient of SIA, due to excess Fe atoms from the deeper layers (while having a vacancy-rich ion-track region).

The work of Pecko et al. [52], on RPV steel JRQ containing 0.15 wt.% of Cu, addressed some empirical connections between Cu atoms and the vacancies introduced by self-ion Fe irradiation (Fe^2+^ 5 MeV; at 300 °C; 2.66 × 10^13^–2.66 × 10^15^). While the vacancy-type defects were found to not be directly responsible for the radiation-induced hardening of the steel, they take part in the radiation-enhanced diffusion of Cu atoms and the formation of Cu-rich clusters.

### 3.2. Hydrogen (Proton) Implantation

In many ways, proton irradiation overcomes the drawbacks of using heavy-ion irradiation as a surrogate for neutron irradiation. Accelerators that are capable of delivering protons of a few MeV are relatively widely available, enabling the modification of tens of micrometer-thick layers in the studied samples by relatively flat damage profiles [1]. The principal benefit of using proton irradiation is the small mass of the proton compared to heavy ions, resulting in a lower energy of recoils. This provides smaller, more widely spaced cascades compared to heavy ions or fast neutrons. Since a relatively small energy of protons is required to overcome the Coulomb barrier, there is often a chance to introduce the activation of certain samples, and this chance increases with proton energy [1].

After losing energy to the electronic degrees of freedom of the metal, a fast proton slows down to thermal velocities and captures an electron to become a hydrogen atom [53]. It is well known that the accumulation of hydrogen can lead to severe embrittlement after reaching a critical concentration near a crack nucleus [1]. Due to the small mass of the proton and the relatively low number of displaced atoms after collision with the target material, the irradiation fluences must be relatively high in order to introduce distinct radiation damage. This opens up the question of the solubility and mobility of hydrogen in the target materials, and the possible effect of blistering. Due to the extremely low activation energy of hydrogen diffusion in alpha iron (0.045 eV), the threshold for blistering in Fe is very high (8 × 10^18^–1.5 × 10^20^ cm^−2^) [54], and ferritic steels can accommodate very high fluences of implanted hydrogen. Another consequence of the high mobility of hydrogen in Fe-based materials is that the Bragg peak is smeared out in-depth by diffusion [54], i.e., the SRIM profiles cannot be simply correlated by the results of, for instance, VEPFIT analysis.

The effect of hydrogen cannot be neglected in any proton irradiation experiments. The diffusion of hydrogen into radiation-induced open-volume defects does not only change the susceptibility of the material to brittle fracture, but it also strongly affects the process of positron trapping at these defects. The estimation of the hydrogen content in the open-volume defects is further complicated by the presence of fast migration paths, such as grain boundaries, lath boundaries, dislocations, etc., which are typical for ferrous alloys and steels.

In the technique of the measurement of Doppler broadening of the annihilation line, the results (line-shape parameters S and W) are often presented in the form of a so-called S-W plot. The linearity of the data in such a plot usually indicates one type of defect in the matrix, with no change in the chemical composition at the vicinity of the annihilation site. Many authors report a change in the linearity (slope) of the S-W data after proton implantation, attributing this trend change to the effect of hydrogen. There are, however, numerous proton irradiation experiments reported with no indication of a change in the S-W trend line. A well-known effect of hydrogen on the lifetime of positrons trapped in vacancy clusters was reported for various materials by theoretical modelling [55,56,57]. The experimental validation of the lifetime data is, however, very complicated for irradiation experiments, leading to the formation of vacancy clusters, which practically involves all types of irradiations except electron and low-energy proton irradiation (which do not lead to the creation of defects larger than monovacancies). Despite these hurdles, proton irradiation combined with post-irradiation examination (PIE), using positron annihilation techniques, provides extremely valuable complementary data for the research of the radiation tolerance of nuclear structural materials. Some selected papers utilizing this approach are summarized in Table 2 and discussed below.

An excellent comprehensive study on a high-energy proton-irradiated Fe–9Cr alloy was published by Xu et al. [15]. Compared to the above-referenced studies, the work by Xu et al. involves proton energies above the spallation reaction threshold, inducing additional transmutation-based hydrogen and helium production, and spallation neutron damage. The positron lifetime of 137.7 ps that was measured for the unirradiated sample was attributed to dislocations and complex defects (vacancies trapped by dislocations), as well as to a carbide component. After irradiation, the long lifetime actually decreased, which was interpreted by the trapping of helium and hydrogen atoms on the above-mentioned defects. A similar reduction in the yield stress and tensile strength, i.e., softening of the alloy, was also explained by interstitial helium (150 MeV irradiation) and hydrogen atoms (11 and 150 MeV irradiations), as well as by the annihilation of jogs on dislocations (11 MeV). Only a few studies involving a proton implantation-induced nuclear reaction (spallation neutron production), and substantial production of transmutation hydrogen and helium in the irradiated samples, were published in the past, employing positron annihilation techniques [37,64,65]. However, as the overview of spallation neutron sources is not the subject of this review, this area of irradiation experiments is not discussed herein in more detail.

Horodek and Kulik used the variable-energy slow positron beam technique of Doppler broadening spectroscopy to characterize proton-implanted pure polycrystalline Fe [58]. Their results provide a good reference for more complex materials, such as alloys and steels modified by proton beams. Using the VEPFIT software, the authors obtained a positron diffusion length, in the reference material, of 158 ± 3 nm. This diffusion length is drastically reduced by proton implantation, to a few nm, regardless of the energy and fluence used in the experiment (see Table 2). The performed VEPFIT analysis further showed that the quasi-homogeneous profile obtained by multi-energy proton implantation can be effectively described by a two-layer model characterizing the implanted region and the unimplanted bulk (substrate) layer. More surprisingly, the two-layer VEPFIT model described single-peak implantation very well using an energy of 73 keV or 173 keV, respectively [58].

Proton-implanted samples of modified 310S steels were characterized by means of slow positron Doppler broadening spectroscopy and transmission electron microscopy in the study by Zhang et al. [59]. Irradiation at 290 °C, using 50 keV H^+^ ions to fluences of 4.0 × 10^16^ and 1.2 × 10^17^ cm^−2^ led to a more pronounced production of hydrogen–vacancy clusters (referred to as proton–vacancy clusters) in Nb-, Ta-, and W-added steels, compared to Zr-added steels. Supported by TEM results on the size of dislocation loops, the authors concluded that the addition of oversized solute Zr to 310S steel leads to a more effective radiation resistance improvement than the addition of Nb, Ta, and W.

An interesting comparison of the proton-implanted samples of German RPV weld materials with their neutron-irradiated counterparts was published by Pecko et al. [60]. Identical levels of proton and neutron fluences were proposed in this experiment, but the relatively low energy of proton implantation resulted in a loss of some information from the conventional ^22^Na positron lifetime measurements, with a positron stopping profile that was well below the introduced damage peak. Nevertheless, the increase in the positron lifetime was found to be roughly the same in the two irradiation experiments (slightly more pronounced for the proton-implanted samples). According to Pecko et al., neither of the irradiation experiments resulted in the formation of large vacancy clusters, with possible consequences in the form of embrittlement of the materials. Again, it is important to note that the use of the conventional radioisotope positron source for the 100 keV proton-implanted samples could not provide a sufficient signal from the implanted layer, and the data were, to a large extent, blurred by the positron annihilation in the unirradiated bulk.

In order to understand the nature of the hardening after radiation in reactor vessel steels, Chinese A508-3 steels were implanted by protons, and investigated by SPB DBS and nanoindentation techniques [61]. A good correlation observed between the two experimental techniques indicates a certain level of retention of hydrogen in the implanted samples, in the form of hydrogen–vacancy complexes, affecting the mechanical properties of the irradiated steel. On the other hand, the obtained S-W plot does not indicate notable changes in the data after proton irradiation, suggesting that there are changes in the nature of the defects. It is important to note that the implantation energy of the H^+^ ions in [61] was 240 keV, in contrast to the 50–173 keV reported in [58], which resulted in most of the positron annihilation data obtained from the region being well below the ion accumulation peak.

The microstructural evolution induced by thermal ageing and proton irradiation was studied by transmission electron microscopy and positron annihilation spectroscopy in the work of Zhao et al. [62]. The study shows that proton irradiation leads to higher densities and larger sizes of dislocation-type defects, but less vacancy-type defects in the T92 steel samples aged at 650 °C (15,000 h) compared to the normalized and tempered reference steel samples. An interesting finding of the experiments was the linearity of the S-W plot, which was more affected by thermal ageing than by proton irradiation. This suggests the creation of some new phases, such as precipitates changing the chemical environment in the vicinity of the annihilation site.

Proton irradiation of structural materials, using energies of 400 keV and more, opens up the potential of applying conventional radioisotope sources as an alternative to slow positron beams. In the study by Ma et al. [63], relatively high fluences of H^+^ ions (1.07 × 10^17^–5.37 × 10^17^ cm^−2^) were used to produce radiation defects in AP1000 reactor containment steel. Although the proton beam modified layer was clearly less than 3 μm thick, an increase in the positron lifetime was observed and used in the complementary characterization, along with TEM. The reason for the observed contrast in the positron lifetime spectra is likely the presence of large voids of several nm, identified by the TEM technique. These can act as strong sinks for positrons, providing a long lifetime component in the lifetime spectra (although the corresponding intensity could be relatively low).

### 3.3. Helium (Alpha) Irradiation

Helium has a detrimental effect on the evolution of radiation damage in steels, with a severe consequence for fission, fusion, and, particularly, accelerator-driven systems (ADS). Many radiation damage studies have been published on nuclear materials, involving helium (alpha) irradiation and various irradiation, and PIE experiments.

The argument against single-beam helium implantation is often the ratio of helium number density to displacement damage (dpa). The helium production rates in some fission applications are well below 1 appm/dpa [66]. In fusion applications, the ratio is on the level of a few tens of appm He/dpa [67], while, in spallation neutron sources, the helium production rates can reach 100 appm/dpa [68]. On the other hand, direct helium implantation (hundreds of keV ion energy) leads to a damage profile with a minimum level of a few hundreds of He appm/dpa, extending to a maximum of a few at.%/dpa. In our earlier study [69], we concluded, however, that the effect of the helium production rate (c_He_/dpa) is not very significant at a helium concentration below a few thousand He appm. A comparison of helium-implanted f/m steels and spallation neutron source-irradiated f/m steels indicated the similar size of cavities (likely helium–vacancy clusters) at similar helium concentrations in the irradiated material.

**Figure 7:**
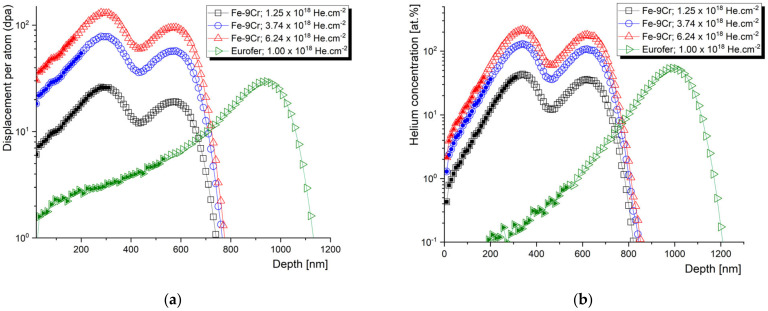
SRIM calculated displacement damage (**a**) and helium concentration (**b**) profiles proposed for slow positron lifetime experiments.

The slow positron beam data obtained from the “flat” part of the helium implantation profile are widely considered as relevant for fusion and spallation applications, while, moving on to the helium concentration peak, the information obtained from the depth profiled measurements becomes devalued by an extremely high helium concentration and broadening of the positron stopping profile. Let us, nevertheless, look at an example of high-fluence helium implantation of the Fe–9Cr alloy, studied by the slow positron lifetime technique. Two similar materials were irradiated by helium, either in single-energy (250 keV) or subsequential two-energy (250 + 100 keV) implantation, to doses ranging from 1 × 10^18^ to 6.2 × 10^18^ cm^−2^ (Figure 7).

**Figure 8:**
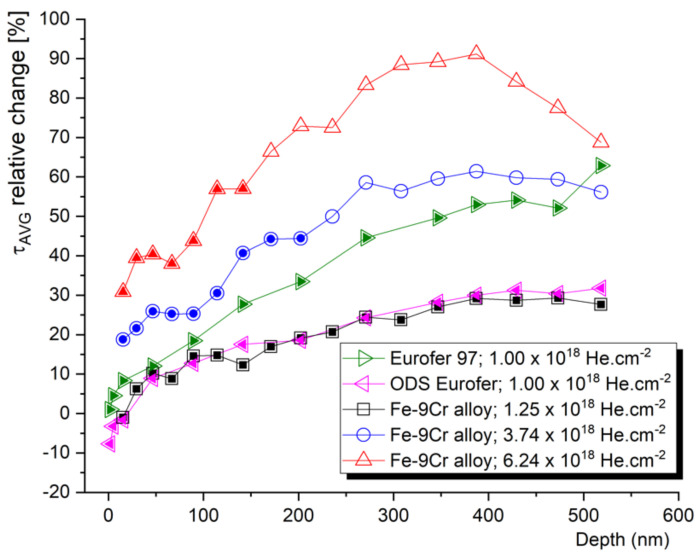
The increase in positron lifetime (implanted–unimplanted) as a function of the mean positron stopping depth. Filled symbols represent data with <50 at.% He, considered in further evaluations.

Despite their low added value, most of the authors almost exclusively perform the evaluation of the slow positron lifetime data by plotting them as a function of the depth of the sample. Let us compare the information given by the positron lifetime depth profiles with the same data evaluated with respect to the irradiation conditions. The average positron lifetime, as the, statistically, most reliable parameter, is plotted as a function of the mean positron stopping depth in Figure 8. These data can be correlated to the SRIM profiles in Figure 7. Despite very different damage and c_He_ profiles, two Fe–9Cr alloys, with very distinct microstructures (Fe–9Cr binary and ODS Eurofer97), show almost identical depth profiles of the average positron lifetime (τ_avg_) change (max. increase of ~30%) when irradiated to fluences of 1.0 × 10^18^ cm^−2^ and 1.25 × 10^18^ cm^−2^, respectively. On the contrary, the positron lifetime increases much more significantly in the non-ODS Fe–9Cr alloy (Eurofer97) implanted by He to 1.0 × 10^18^ cm^−2^ (max. increase of ~55%). With increasing the total He fluence to 3.7 × 10^18^ cm^−2^ and 6.2 × 10^18^ cm^−2^, the average positron lifetime in the binary Fe–9Cr alloy increases by 60% and 90%, respectively. In the first approach, we must consider three factors affecting these profiles. The first is the presence of defects sinks (such as grain boundaries, lath boundaries, dislocations, internal surfaces, etc.) in the pristine material. The second is the formation of new defect sinks (such as cavities) by agglomerations of radiation-induced defects. These cavities become primary sinks for new point defects and helium ions at high concentrations, and significantly suppress further growth of the radiation-induced cavities. Finally, the increase in positron lifetime in the given irradiated materials naturally depends on the fluence (number density) of the implanted helium ions.

The depth profile of the average positron lifetime, such as the one in Figure 8, often causes scientists to incline to correlate it with SRIM profiles. This is, however, quite meaningless above a certain level of helium fluence/concentration, due to the accumulation of helium, blistering, and following exfoliation of the ion beam modified layer. Given the two energies of helium implantation used and the performed scanning electron microscopy (SEM) analysis, we can conclude that the blistering threshold for helium in the Fe–9Cr alloy is somewhere between 1.3 and 3.5 × 10^18^ cm^−2^. At a total He fluence of 3.74 × 10^18^ cm^−2^, about 15% of the surface was exfoliated, while at the fluence of 6.24 × 10^18^ cm^−2^, about 25% of the surface was exfoliated after the implantation. The relative change (increase) in positron lifetime was multiplied by 1.15 and 1.25, respectively. The reason for this was to provide a more accurate estimation of positron lifetimes in extremely damaged Fe alloys. Nevertheless, in further evaluation, the peak concentration data were not considered, and irradiation conditions with a maximum of 40–50 at.% of helium were plotted in further graphs. These ranges of damage profiles are indicated by full symbols in Figure 7 and Figure 8.

**Figure 9:**
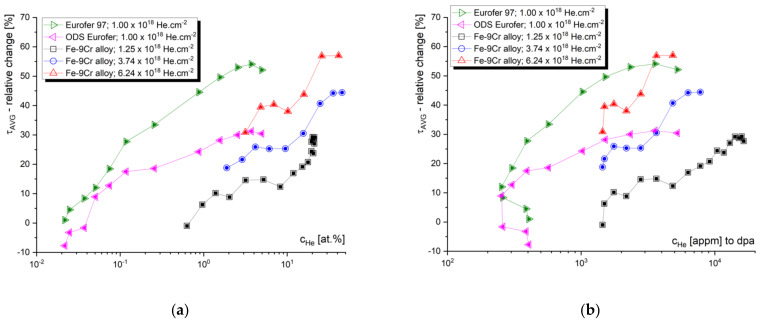
Relative increase in the average positron lifetime as obtained for the investigated samples. Data are plotted as a function of helium concentration c_He_ (**a**) and as a function of helium/dpa ratio (**b**).

Figure 9 shows the relative values (implanted/un-implanted) of the average positron lifetimes obtained for the helium-implanted samples, and plotted as a function of c_He_ and c_He_/dpa, respectively. In contrast to slow positron lifetime data evaluated with respect to the (mean) stopping depth of the positron, one can observe specific irradiation conditions leading to the given positron lifetime change. Although the number density of helium (c_He_) seems to be the most representative irradiation parameter, it is important to not detach the c_He_ from the displacement damage (dpa). Since different energy of incident ions leads to a different number of primary knocked-on atoms (PKA), the information of displacement damage must be considered in the data interpretation. Figure 9 suggests that the increase in positron trapping at the defects increases rapidly at lower helium concentrations (lower dpa), and this increase slows down near saturation at high helium concentrations. It is reasonable to assume that this saturation is caused by new radiation-induced sinks for point defects and helium. At low c_He_, the primary sinks for radiation-induced defects are the initial lattice defects (such as grain boundaries, lath boundaries, dislocations, internal surfaces, etc.), while at certain He fluences, the sinking of point defects takes place primarily at helium bubbles and other types of radiation-induced cavities. This must be considered in the experiments leading to the production of a high density of such cavities, i.e., effective defect sinks, which can give a certain impression of good radiation resistance.

**Figure 10:**
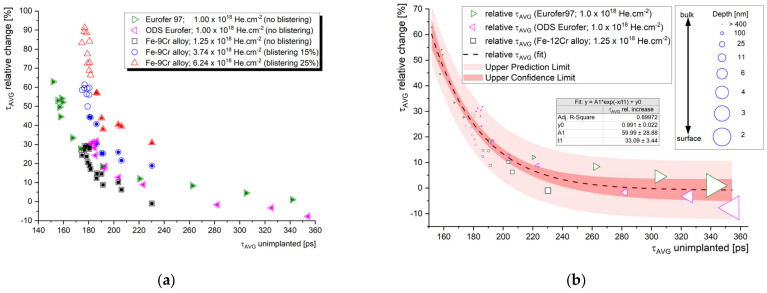
Relative change in the average positron lifetime in different irradiation experiments plotted as a function of the corresponding lifetime of the unirradiated sample (**a**). Exponential decay fit of datapoints obtained for three different Fe–9Cr alloys irradiated to He fluences ≤1.25 × 10^18^ cm^−2^ (**b**).

Although τ_avg_ is the most reliable parameter statistically and its variation can be used for a rough assessment of the radiation tolerance of a given material, the comparison of absolute positron lifetime values between different materials and/or different irradiation conditions is far from being straightforward. It is, however, the aim of this paper to provide some empirical suggestions for researchers, regarding the interpretation of experimental PAS data in the research of the radiation resistance of materials. Let us plot the relative τ_avg_ data from Figure 9 as a function of the corresponding average lifetime obtained for unirradiated material (τ_avg_ un-implanted). As shown in Figure 10, such a relationship is fairly similar for the three Fe–9Cr alloys studied, which were irradiated to a similar fluence (1–1.25 × 10^18^ cm^−2^). As expected, the most pronounced change (increase) in the positron lifetime was obtained for measurements where the τ_avg_ of the un-implanted sample was the shortest. In other words, the presence of trapping sites for positrons in the unirradiated material can be correlated with the presence of sinks for radiation-induced defects, i.e., the potential for local defect recombination. It is not surprising that the high positron lifetimes of the SPB data correspond to the surface and near-surface region (Figure 10b). This underlines the importance of considering the “surface effect” (positron diffusion to the surface), which acts both as a strong positron trap, and a sink for radiation-induced defects and helium.

Based on Figure 10, we can conclude that a comparison of the average positron lifetime in unirradiated materials can provide a useful estimation of the potential radiation tolerance of the material in the swelling incubation phase. In other words, the effectiveness of the initial defect sinks in as-received material can be conveniently predicted using positron lifetime spectroscopy. This technique is also capable of tracking the microstructural evolution in the bubble or void swelling regime, i.e., when the fluence increases to the point where new internal surfaces emerge in the material bulk, such as large cavities or blisters. In such conditions, the interpretation of PAS data can be supported by, and correlated to, TEM.

In addition to the positron annihilation lifetime measurement of helium-implanted metals, valuable complementary information on the chemical composition at the vicinity of the annihilation site can be obtained by DBS. In our earlier work [70], theoretical calculations of ratio profiles for empty and He-filled vacancy clusters were performed to investigate the possibility of direct He detection in radiation-induced open-volume defects, by means of the DBS technique. The comparison of theoretical momentum profiles for monovacancy and nine-vacancy clusters, with and without the presence of contained helium atoms suggests that a characteristic helium-related peak can be expected in the momentum range of 5–7 × 10^−2^ m_0_c. By comparing experimental momentum profiles of the He-implanted Fe–12Cr alloy obtained from the ion track region (low concentration of implanted He ions) and He peak region, we reported experimental evidence of the anticipated He peak. This was later confirmed by Sabelova et al. [71], on annealed Eurofer97 steel irradiated in a spallation neutron source. Although a direct experimental confirmation of a helium signal in the (C)DBS spectra is usually complicated by various factors (mostly the complexity of the material microstructure), the technique is, without any doubts, a very effective tool for the investigation of helium behavior in irradiated structural materials.

In the work of Sharma et al. [72], DBS measurements of He-implanted Ni–Cr alloys have been conducted, along with comprehensive theoretical calculations of positron lifetime characteristics in this type of alloy. These calculations confirm the findings of previous studies on the effect of helium on positron lifetime in vacancy clusters. Similarly to the earlier reported work of Troev et al. [57], the study by Sharma et al. reports a significant decrease in positron lifetime in vacancy clusters (VC), with an increasing number of implanted helium ions. This effect is slightly more pronounced compared to the same quantity of hydrogen atoms. On the other hand, however, this study shows that with increasing the number of implanted ions surrounding the vacancy defects, the S parameter is decreased, which is attributed to the electron momentum distribution of the helium ions. The authors suggest that in the case of He, 1S and 2S electrons have a very large contribution to the low-momentum region (0–1.8 × 10^−3^ m_0_c). As a result, the S-parameter value drastically increases with the increase in He atoms surrounding the vacancy defects. This calculation is practically in agreement with the calculations of the characteristic helium peak by Sabelova [70], which indicated that the position of the peak was somewhere in between the regions generally attributed to the S and W parameters. In the experimental part, Sharma et al. reported that the relative density of isolated vacancy defects in the cascade (damage peak) region was smaller compared to the ion track region in the investigated He-irradiated alloys.

Somewhat higher momentum values (8–14 × 10^−3^ m_0_c) were attributed to the helium peak in the CDBS spectra of the Fe–9Cr alloy irradiated by He ions at 550 °C, in the work of Zhu et al. [73]. In this work, the authors investigated the effect of irradiation temperature on the formation of helium–vacancy clusters. The reported results suggest a coexistence of large amounts of He_m_V_1_ and monovacancies in the sample irradiated at room temperature. However, irradiation at a temperature between 250 and 550 °C leads to the absorption of helium atoms by helium–vacancy clusters, and the formation of over-pressurized He_m_V_n_ (m > *n*) clusters or helium bubbles. The results also show that void swelling in the investigated Fe–9Cr alloy is most pronounced under 450 °C irradiation.

Fe–9Cr steel Eurofer 97, together with its ODS variant, implanted by 500 keV helium ions, was investigated using the SPB DBS technique and reported in our recent work [74]. Using VEPFIT analysis, we characterized the positron diffusion in the studied materials and determined the effective positron diffusions length. As expected, this length is significantly shorter in the ODS materials (5 nm in the ODS Eurofer vs. 35 nm in as-received Eurofer97), and the sample preparation plays an important role in the evolution of the radiation-induced point defects in the near-surface region.

The near-surface region of helium-implanted Eurofer97 steel was reported earlier by Carvalho et al. [75]. In this study, extraordinarily low energy of implanted helium ions (30 keV) was used in the irradiation experiments, providing a damage peak at a depth of ~20 nm. Despite the likely strong sinking of the created defects by the sample surface, the results show a clear effect of the initial microstructure on the evolution of helium–vacancy clusters. In the as-received (polished) samples, a decrease in the S parameter was observed after implantation, while no significant change in the S parameter was observed after the implantation of annealed samples. This was explained by helium absorption by pre-existing vacancies and clusters of vacancies, which dominated the creation of new defects. Similar conclusions were obtained for the early stage of the spallation neutron source irradiation of the Fe–9Cr alloy, and published in the paper by Krsjak et al. [37]. The paper by Carvalho et al. further confirms that an irradiation temperature of 100 °C leads to the formation of small helium–vacancy clusters, while large helium bubbles are being formed already at 250 °C. In this regard, the Doppler broadening data are in good agreement with the thermal desorption spectroscopy (TDS) results.

Chinese low-activation martensitic steel CLAM was investigated after 140 keV He implantation by DBS and nanoindentation techniques, in the work by Xin et al. [76]. The work concludes that the concentration of vacancy-type defects decreases with increasing irradiation temperature, ranging from RT to 600 °C. The results of nanoindentation show that the irradiation-induced hardening, observed at all irradiation temperatures, is most pronounced at 200 °C. This suggests that both vacancy–helium complexes and helium bubbles substantially contribute to irradiation-induced hardening.

The behavior of open-volume defects in stainless steel 316L was investigated by the DBS technique, as a function of post-irradiation annealing temperature, by L. Song et al. [77]. The authors reported competition between the formation of helium–vacancy clusters and the recombination of radiation-induced vacancies in the temperature range 100–400 °C. Above 500 °C, a steady-state growth of helium bubbles was observed. After the annealing temperature was increased to above 800 °C, unstable He_n_V_m_ clusters were observed to dissociate into vacancies and He atoms, and eventually disappear. This was observed via a decrease in the S parameter in the near-surface region, as the vacancies sank to the surface and the He atoms diffused out of the sample.

The same stainless steel (316L), and an Fe16.7Cr14.5Ni model alloy, were studied by DBS after irradiation by 140 keV He ions at different fluences, by Lu et al. [78]. In addition to room-temperature irradiation, the investigated samples were irradiated at 300 °C. The authors reported (He-free) vacancy-type defects as a major defect type in the ion track region, while the main defects in the cascade region were identified as He–vacancy complexes. Only small differences were observed between the microstructural changes in the two studied materials after room-temperature irradiation, while irradiation at 300 °C led to more pronounced differences. The much more dramatic increase in the S parameter in the model alloy was explained by the effect of microelements in 316 SS. The authors confirmed previous studies suggesting that impurities, such as C, N, H, and He, can potentially increase the vacancy migration energy in Fe-based alloys.

The irradiation conditions and the used experimental techniques reviewed in this section are summarized in Table 3 below.

### 3.4. Synergistic Effects of Helium and Hydrogen on Self-Ion-Induced Damage in Steels

The production of transmutation helium in structural materials exposed to harsh radiation environments rarely occurs without accompanying production/accumulation of hydrogen. Whether it is fusion or spallation application, structural materials suffer from synergistic effects of hydrogen and helium accumulated in the microstructure of the irradiated material. One of the great opportunities to study these synergistic effects is spallation neutron target irradiation. With a helium production of ~75–90 appm/dpa and H production of ~300–400 appm/dpa, spallation neutron target irradiation provides unique irradiation conditions for such a study. On the other hand, the production rate of the transmutation elements is proportional to the proton/neutron flux, which naturally determines the irradiation temperature gradient. It is, therefore, very difficult to investigate any standalone irradiation parameter and its effect on the microstructure in spallation neutron target irradiation.

Numerous studies on the experiments were published recently, using sequential or simultaneous hydrogen, helium, and self-ion implantation in a wide range of fluences and irradiation or post-irradiation annealing temperatures. The convenient control of the irradiation parameters enabled various effects to be studied independently, which provided a unique set of data for improving the knowledge in this challenging area of nuclear material research.

One of the first studies utilizing slow positron beam experiments on sub-sequential H- and He-implanted samples was published on CLAM steel by Xin et al. [79]. In reference to unirradiated material, a positron diffusion length of 120 ± 8 nm was obtained by VEPFIT analysis. This is a relatively high value for martensitic steel, and suggests a rather low concentration of positron traps in pristine material. The value of the S parameter in the samples pre-implanted by H was found to be higher compared to the samples pre-implanted by He. This difference decreased with increasing fluence.

At about the same time, Kögler et al. investigated open-volume defect generation and its impact on the hardness of ODS FeNiCr in a dual-beam experiment, applying sequential, as well as simultaneous, Fe and He irradiation [80]. The unirradiated materials contained dislocations and clusters of 4–5 vacancies that were closely related to Y-Al oxide nanoparticles (being less pronounced for samples having undergone heat treatment prior to irradiation), whereas, after irradiation, significant differences were found in the hardness increase for the simultaneous and sequential process in the case of the non-heat-treated sample. This effect was attributed to the immediate stabilization of vacancy clusters by helium during simultaneous irradiation, in contrast to the sequential process, where helium implantation followed Fe. The heat-treated sample, on the other hand, did not exhibit significant differences owing to the irradiation sequence, which, however, might have been caused by a He concentration that was too low. At elevated temperatures, 300 °C, simultaneous dual implantation resulted in a surprisingly large decrease in open-volume defects, and the remaining defects were 3–4 vacancy clusters, in connection with the Y-Al oxide particles, similarly to the untreated sample.

Another Chinese reduced activation ferritic/martensitic (RAFM) steel SIMP, with enhanced high-temperature oxidation and liquid metal corrosion resistance, was investigated by DBS and TEM, after sequential H and He (room temperature) implantation, in a very comprehensive work of Jin et al. [81]. Similarly to the study by Xin et al. [79], the increase in the S parameter of the H-implanted sample was found to be higher than in the case of the He-implanted sample, despite the lower displacement damage introduced by hydrogen implantation. The authors interpreted this by the higher trapping rate of helium atoms by vacancies, compared to H atoms. Helium is expected to occupy the center of vacancies. Any sequential implantation by both He and H ions led to a lower ΔS parameter compared to separate H implantation. The increase in the S parameter He + H was more pronounced compared to the H + He sequence when the hydrogen fluence was sufficiently high. The TEM results indicated that a smaller size and higher density of bubbles are produced in the steel after He + H implantation compared to the hydrogen-only implanted sample. The authors, moreover, conclude that the hydrogen atom prefers the interstitial positions in the vicinity of vacancies and tends to form a H–vacancy complex. Hydrogen is, nevertheless, effectively captured by helium–vacancy clusters, and the existence of such agglomerations leads to the suppression of direct binding between H atoms.

The effectivity of helium–vacancy nano-clusters in storing hydrogen atoms was investigated in detail in work by Zhu et al. [82]. The authors used first-principle calculations and positron annihilation spectroscopy to study the potential of defect sites, formed after irradiation, for hydrogen storage. The study confirms, again, the effectivity of hydrogen capturing by helium–vacancy clusters or bubbles formed in He implantation.

A study focusing on hydrogen-assisted embrittlement of reactor pressure vessel (RPV) steels was reported by Shi et al. [83]. The authors used proton and Fe^13+^ ion irradiation to simulate a harsh radiation environment and study relatively (compared to the usual displacement damage in RPV steels) severely damaged samples. The study shows that the S parameter does not saturate for proton-irradiated (A508-3) steel, even at a relatively high displacement damage (2.26 dpa). On the other hand, Fe ion implantation leads to fast S-parameter saturation, and further irradiation does not increase positron trapping at radiation-induced defect agglomerations. It is reasonable to assume that Fe implantation at 3 MeV produces voids that become sinks for new radiation-induced vacancies, and prevents further production of new helium–vacancy agglomerations.

A comprehensive study of RPV steels under proton and ion irradiation has been published by Jiang et al. [84], employing PAS, atom probe tomography (APT), transmission electron microscopy (TEM), and nanoindentation. The authors identified a wide range of defects, from small-size defects, such as vacancies, vacancy–solute complexes, dislocation loops, to large-size vacancy clusters and cavities. The study aimed to investigate the difference between the microstructural evolution of RPV steels irradiated by protons (240 keV) and heavy ions at low temperatures. Although the synergistic effect between the different implanted ions was not investigated, the study reports unique data provided by a combination of many experimental techniques. Similarly to the study by Shi et al. [83], positron trapping at radiation-induced defects was observed to saturate rapidly in Fe ion implantation at a relatively low dose. On the one hand, positron trapping increased without a notable saturation in the samples irradiated by protons. The atom probe did not reveal any solute segregation at low irradiation temperatures. On the other hand, TEM showed the formation of dislocation loops after proton irradiation to 1 dpa, which were responsible for the increase in hardness measured by the nanoindentation technique. The authors suggest that proton irradiation induces the migration of vacancies and their agglomeration into vacancy clusters. These are expected to be stabilized by hydrogen atoms. Fe implantation resulted in a high density of interstitial and vacancy clusters, inducing the formation of dislocation loops and corresponding to the increase in nano-hardness.

Interesting PAS studies were published on different irradiation experiments on Indian reduced activation ferritic/martensitic steel (INRAFM). An isochronal annealing study has been published on INRAFM samples that were individually irradiated with H and He ions, and sequentially irradiated with both H and He ions [85]. Among the single-ion irradiated samples, the hydrogen-irradiated sample showed the presence of hydrogen–vacancy complexes with a high H content in the as-irradiated state, and an increase in the S parameter, due to the release of H from these complexes, at 100 °C. Complete recovery of the microstructure was observed after annealing at 400 °C. On the other hand, three distinct stages of annealing were identified in the helium-only irradiated samples. There was a defect annealing stage, with a decrease in the S parameter from as-irradiated to 300 °C, bubble nucleation stage, with a stable S parameter from 300 °C to 400 °C, and the bubble growth region, characterized by an increase in the S parameter, was observed between 400 °C and 700 °C. It is reasonable to assume that the first stage is affected by the diffusion of helium into existing sinks (open-volume defects), which naturally reduces the rate of positron trapping at these defects. Similar behavior was observed, for instance, in the work of Sabelova et al. [71]. The authors concluded that when sequential H and He implantation is used, the nucleation and growth of helium bubbles, due to isochronal annealing, are independent of the irradiation sequence, and this is not affected by the presence of hydrogen.

Another ion irradiation experiment involving DBS characterization has been published on the Indian INRAFM steel in [86]. The samples were irradiated up to 70 dpa by Fe ions at different temperatures, with and without helium (~700 appm) pre-implantation. The DBS data confirmed that radiation-induced vacancy-type defects can be effectively annealed at high-temperature Fe irradiation. Complete recovery from irradiation-induced vacancy-type defects was observed at 400 °C. The sample pre-implanted by helium also exhibited a decrease in the S parameter with an increasing irradiation temperature up to 400 °C, but this was due to helium absorption by vacancy clusters and an increase in the He/V ratio. At a higher irradiation temperature, the formation and growth of helium bubbles was observed by a notable increase in the S parameter. Again, this observation is in good agreement with other PAS studies of the irradiation/annealing temperature dependencies of positron trapping in samples containing injected or transmutation helium.

A different approach to the study of the synergistic effect of hydrogen and helium was used in the work of Li et al. [87]. In this study, the authors used Ni^+^ pre-implantation to produce radiation-induced vacancy clusters in the studied samples of the Fe–9Cr alloy. Such samples were consequently irradiated by H and He ions separately or subsequently. The study showed that the S parameter after sequential He + H implantation was almost identical to the S parameter obtained for the samples irradiated by only He ions. The authors concluded that H atoms implanted into He pre-implanted material can be effectively absorbed by He_n_V_m_ clusters by forming He_n_V_m_H complexes. The authors confirmed that helium ions implanted into H pre-implanted material might decompose H_n_V_m_ clusters and release hydrogen into the matrix. This can be accompanied by a production of additional vacancy defects.

Excellent work by Scepanovic et al. [88] was published on the Fe–14Cr alloy and its ODS variant, subjected to single Fe^+^ or He^+^ ion irradiations at different doses and temperatures. All the experimental techniques applied, namely, PALS, DBS, and TEM, confirmed a more pronounced production of radiation-induced defects in the non-ODS alloy. The experiments confirmed the role of Y-rich nanoparticles in acting as vacancy sinks, hindering the growth of helium bubbles. The published results indicate that positron traps after the irradiation of the ODS alloy are not associated with nanoparticles, but rather with the ferritic matrix. Similarly, TEM data suggested that helium bubbles are mostly associated with the ferritic matrix in both materials, although the coarsening of the bubbles was clearly hindered in the ODS alloy.

Also of interest is another study of this group on Fe–15Cr steel, where the influence of external magnetic fields on defect evolution was studied and found to be “not insignificant” [89]. In the presence of a B field, the samples show a lower vacancy density and smaller cluster sizes compared to irradiation in the absence of it, yet the significance of this effect will be investigated in the future.

One of the few PAS studies utilizing simultaneous implantation, triple-ion (Fe^+^, He^+^, H^+^) irradiation has been published by Parente et al., on the Fe–12Cr alloy (including its ODS variant) and two different ODS Fe–14Cr alloys [16]. The reported DBS momentum profiles suggest that the defects induced by this irradiation in the ODS Fe-12Cr and non-ODS Fe12Cr alloys appear to have the same structural characteristics as Cr atoms, as they are the nearest neighbors. The authors pointed out that defects with similar characteristics were also present in the unirradiated materials produced by powder metallurgy. The S-parameter curves obtained on the ODS Fe–14Cr alloy samples, irradiated by the triple beam at 600 °C, were found to be depth independent for positron energies >5 keV. The obtained S-parameter values were slightly lower than the corresponding values for the unirradiated alloy. This was explained by the trapping of H^+^ and He^+^ at the pre-existent vacancy clusters. The CDB spectra of the irradiated ODS Fe–14Cr samples, normalized to the corresponding unirradiated sample, pointed out the association between the new radiation-induced vacancy clusters and Cr atoms.

The irradiation conditions and the used experimental techniques reviewed in this section are summarized in Table 4 below.

## 4. Summary

Over the past two decades, positron annihilation spectroscopy has proven to be an invaluable characterization tool in various radiation damage studies aimed at the evolution of early-stage microstructures. However, due to the wide variety of materials and irradiation conditions to be studied, and the complexity of data analysis, it is often difficult to reflect the knowledge obtained so far in new irradiation studies. This paper summarizes recent studies of structural materials subjected to different ion beam irradiation experiments and investigated by techniques of positron annihilation spectroscopy. It provides a convenient reference for experiment proposals in the field and enables a quick comparison of the obtained results to the published data. This helps researchers to conduct more reproducible experiments, and will eventually help them to use positron annihilation spectroscopy as a primary characterization tool.

## Figures and Tables

**Table 1:** Selected PAS studies published on self-ion irradiation of nuclear structural materials.

Material	Ion (E)	T [°C]	Fluence(s) [cm^−2^]	Techniques	Ref.
FeCrCoNi (HEA)	Ni^+^ (1.5 MeV)	500	5 × 10^16^	DBS (SPB 0.25–21.5 keV) + VEPFIT analysis; GIXRD; EBSD	[47]
Fe film	Fe^+^ (2 MeV)	RT	5.65 × 10^14^	DBS (SPB 0.05–16 keV) + VEPFIT analysis; LT (SPB 0.5–16 keV)	[48]
BCC Fe	Y^+^ (1.2 MeV)	RT	1.0 × 10^14^ 2.0 × 10^15^ 3.0 × 10^15^	DBS (SPB 0.5–25 keV) + VEPFIT analysis, SIMS	[49]
Fe–9%Cr (T91 steel)	Fe^+^ (3.25 MeV)	RT; 300; 450	1.7 × 10^16^	DBS (SPB 0.5–20 keV) + VEPFIT analysis; nanoindentation	[50]
High Si Fe–11Cr	Fe^+^ (3.25 MeV)	RT, 450	4.3 × 10^15^; 1.7 × 10^16^	DBS (SPB 0.18–20.18 keV) + VEPFIT analysis	[51]
RPV steel (JRQ)	Fe^2+^ (5 MeV)	300	2.66 × 10^13^ - 2.66 × 10^15^	DBS (SPB 30 eV–36 keV); Nanoindentation	[52]

**Table 2:** Selected PAS studies published on proton irradiation of nuclear structural materials.

Material	Ion (E)	T [°C]	Fluence(s) [cm^−2^]	Techniques	Ref.
Fe–9Cr alloy	H^+^ (11 MeV); H^+^ (150 MeV), incl. H (1100 appm/dpa) and He (120 appm/dpa)	RT (150 MeV); 300 (11 MeV)	4.5 × 10^15^ (150 MeV); 6.0 × 10^16^ (11 MeV);	PALS (^22^Na); Tensile	[15]
Polycrystalline Fe	H^+^ (73 keV; 173 keV; multi-energy 50–173 keV)	RT	3 × 10^15^; 1 × 10^16^; 3 × 10^16^;	DBS (SPB 0.1–36 keV) + VEPFIT analysis; Elastic Recoil Detection (ERD)	[58]
Modified 310S steel	H^+^ (50 keV);	290	4.0 × 10^16^; 1.2 × 10^17^;	DBS (SPB ~ 0.2–20 keV); TEM	[59]
German RPV weld metals	H^+^ (100 keV)	<100	6.2 × 10^17^; 5.1 × 10^18^; 2.0 × 10^19^;	PALS (^22^Na)	[60]
Chinese RPV steel (A508-3)	H^+^ (240 keV)	<100	2.5 × 10^16^; 5.5 × 10^16^; 1.1 × 10^17^; 2.5 × 10^17^;	DBS (SPB 0.25–26 keV); Nanoindentation	[61]
Fe–9%Cr steel (T92)	H^+^ (250 keV)	RT	0.01 dpa; 0.05 dpa; 0.2 dpa	DBS (SPB 1–25 keV); TEM; Nanoindentation	[62]
SA-738Gr.B steel (AP1000 reactor containment steel)	H^+^ (400 keV)	150	1.07 × 10^17^; 2.68 × 10^17^; 5.37 × 10^17^;	PALS (^22^Na);TEM	[63]

**Table 3:** Selected PAS studies published on helium irradiation of nuclear structural materials.

Material	Ion (E)	T [°C]	Fluence(s) [cm^−2^]	Techniques	Ref.
Fe–12%Cr alloy	He (250 keV)	<80	1.25 × 10^18^	LT (SPB 1 to 18 keV) DBS (SPB 0.5 to 38 keV)	[70]
Ni–40% Cr alloy	He (65 keV)	-	3 × 10^16^; 2 × 10^17^; 7 × 10 ^17^	DBS (SPB 0.2–20 keV) + VEPFIT analysis; Theoretical calculations	[72]
Fe–9%Cr alloy	He (100 keV)	250; 350; 450; 600	5 × 10^16^	PALS (^22^Na); DBS (SPB ~0.1 to ~20 keV); CDBS (SPB 8 keV)	[73]
Fe–9%Cr–1%W steel (Eurofer97) + its ODS variant	He (500 keV)	RT	1 × 10^18^	DBS (SPB 0.5 to 36 keV)	[74]
Fe–9%Cr–1%W steel (Eurofer97)	He (3 keV)	100; 250	3 × 10^19^ (100 °C) 1–2 × 10^19^ (250 °C)	DBS (SPB 0.1 to 25 keV); TDS	[75]
Fe–9%Cr steel (CLAM)	He (140 keV)	RT–600	1 × 10^16^	DBS (SPB ~0.1 to ~21 keV) + VEPFIT analysis; Nanoindentation	[76]
316L stainless steels	He (50 keV)	RT (post-irr. anneal. 100–1000)	1 × 10^16^	DBS (SPB 0.18 to 20 keV)	[77]
FeCrNi alloy 316L stainless steels	He (140 keV)	RT; 300	1 × 10^16^ (RT); 5 × 10^16^ (RT; 300 °C)	DBS (SPB 0.18 to 20 keV)	[78]

**Table 4:** Selected PAS studies published on synergistic effects of heavy-ion and H/He irradiation on the microstructure of nuclear structural materials.

Material	Ion (E)	T [°C]	Fluence(s) [cm^−2^]	Techniques	Ref.
Fe–9%Cr steel (CLAM)	H (80 keV,); He (140 keV)	RT	H: 5 × 10^15^–5 × 10^16^ He: 1 × 10^15^–1 × 10^16^	DBS (SPB 0.1–20 keV) + VEPFIT analysis	[79]
ODS FeCrAl (PM2000)	Fe (2.5 MeV) He (350 keV)	RT, 300	2.74 × 10^15^/He 2.94 × 10^16^/Fe	LT (^22^Na) DBS, CDBS (SPB 30 eV–35 keV) + VEPFIT analysis Nanoindentation	[80]
Fe–9%Cr steel (SIMP)	H (80 keV, 260 keV); He (130 keV; 500 keV)	RT	H: 2.78 × 10^15^–1 × 10^18^ He: 7.14 × 10^15^–1.8 × 10^16^	DBS (SPB 0.18–20 keV) TEM	[81]
Fe–9%Cr steel	H (80 keV,); He (140 keV)	RT	H: 5 × 10^16^ He: 1 × 10^16^	LT (^22^Na); DBS (SPB 0.18–20 keV) + theoretical calculations	[82]
RPV steel (A508-3)	Fe (3 MeV) H (240 keV)	100	H: 2.5 × 10^16^–1.13 × 10^18^ Fe: 4.55 × 10^13^–2.06 × 10^15^	DBS (SPB 0.5–26 keV)	[83]
RPV steel (A508-3)	Fe (3 MeV) H (110 + 240 keV)	100	0.05–1.0 dpa;	DBS (SPB 0.5–26 keV) LT (SPB 14 keV); APT; TEM; Nanoindentation	[84]
Fe–9%Cr steel (INRAFM)	Fe (1.1 MeV) He (50 + 90 + 130 keV)	200, 400, 500	Fe (70 dpa) He (700 appm)	DBS (SPB 0.2–22 keV) + VEPFIT analysis	[85]
Fe–9%Cr steel (INRAFM)	H (80 keV) He (130 keV)	RT; Post-irradiation annealing up to 700	H (1500 appm); He (450 appm)	DBS (SPB 0.25–22 keV) incl. VEPFIT analysis	[86]
Fe9Cr alloy	Ni (1 MeV); H (50 keV); He (80 keV)	RT (Ni); RT (Ni + H); RT (Ni) + 450 (H); RT (Ni) +450 (H) 450 (He)	Fe: 3.4 × 10^13^ H: 1 × 10^16^ He: 2 × 10^15^	DBS (SPB 0.18 keV–20.18 keV)	[87]
Fe14Cr alloy; Fe14Cr ODS	Fe (1 MeV); He (50 keV);	RT (Fe) 400–450 (He)	Fe: 6.5 × 10^15^ He: 6.5 × 10^15^; 1 × 10^16^	DBS (SPB ~0.1 keV–30 keV) CDBS (SPB 7 keV; 18 keV)	[88]
Fe15Cr alloy	He (45 keV) Fe (1 MeV)	RT	He: 4.0 × 10^16^ Fe: 8.24 × 10^15^; 1.12 × 10^16^	DBS, CDBS (SPB ~0.2 keV–30 keV)	[89]
Fe12Cr ODS12Cr ODS14Cr ODS14CrWTi	Fe (10 MeV); Fe (4 MeV)/ He (1.6 MeV)/ H (500 keV)	RT (Single beam); 600 (Triple beam)	Fe: 5 × 10^15^; Fe: 1.49 × 10^16^/He 1.4 × 10^15^/H 3.55 × 10^15^	DBS (SPB 1–30 keV) CDBS (SPB 30 keV)	[16]
